# From Diabetic Nephropathy to End-Stage Renal Disease: The Effect of Chemokines on the Immune System

**DOI:** 10.1155/2023/3931043

**Published:** 2023-05-30

**Authors:** Yuheng Qiu, Jingyi Tang, Qihan Zhao, Yuhua Jiang, Yu Ning Liu, Wei Jing Liu

**Affiliations:** ^1^Dongzhimen Hospital, Beijing University of Chinese Medicine, Beijing, China; ^2^Renal Research Institution of Beijing University of Chinese Medicine, Key Laboratory of Chinese Internal Medicine of Ministry of Education and Beijing, Dongzhimen Hospital Affiliated to Beijing University of Chinese Medicine, Beijing, China

## Abstract

**Background:**

Diabetic nephropathy (DN) is a major cause of end-stage renal disease (ESRD), and there is growing evidence to support the role of immunity in the progression of DN to ESRD. Chemokines and chemokine receptors (CCRs) can recruit immune cells to sites of inflammation or injury. Currently, no studies have reported the effect of CCRs on the immune environment during the progression of DN to ESRD.

**Methods:**

Differentially expressed genes (DEGs) from the GEO database were identified in DN patients versus ESRD patients. GO and KEGG enrichment analyses were performed using DEGs. A protein-protein interaction (PPI) network was constructed to identify hub CCRs. Differentially expressed immune cells were screened by immune infiltration analysis, and the correlation between immune cells and hub CCRs was also calculated.

**Result:**

In this study, a total of 181 DEGs were identified. Enrichment analysis showed that chemokines, cytokines, and inflammation-related pathways were significantly enriched. Combining the PPI network and CCRs, four hub CCRs (CXCL2, CXCL8, CXCL10, and CCL20) were identified. These hub CCRs showed an upregulation trend in DN patients and a downregulation trend in ESRD patients. Immune infiltration analysis identified a variety of immune cells that underwent significant changes during disease progression. Among them, CD56bright natural killer cell, effector memory CD8 T cell, memory B cell, monocyte, regulatory T cell, and T follicular helper cell were significantly associated with all hub CCR correlation.

**Conclusion:**

The effect of CCRs on the immune environment may contribute to the progression of DN to ESRD.

## 1. Introduction

Diabetic nephropathy (DN), a common complication of diabetic microangiopathy, is a major cause of end-stage renal disease (ESRD) with significant morbidity and mortality [1, 2]. DN is reported to account for 50% of new cases of ESRD each year, increasing the incidence of ESRD by approximately 12-fold and the overall mortality rate by nearly 20% [3–5]. Between 2003 and 2017, more than 220,000 deaths were attributed to advanced CKD/ESRD in the United States, and even when patients have been treated, 25% of patients with type 2 diabetes and DN will eventually progress to ESRD [6]. The underlying mechanism why DN progresses more rapidly to ESRD and has a poorer prognosis is still unclear. Therefore, it is crucial to explore the biological mechanisms of progression from DN to ESRD and to find reliable biomarkers.

Chemokines are a family of small secreted proteins that bind to chemokine receptors and direct the migration of immune cells to sites of inflammation or injury by triggering intracellular signaling pathways [7]. Inflammation is recognized as a key factor driving the pathogenesis and maintenance of DN [8–10]. When mesenchymal stem cells are exposed to the inflammatory environment of DN, they release chemokines to coordinate local and systemic immune responses [11]. Although DN is not considered an immune-mediated disease, increasing evidence supports the role of innate and adaptive immunity in the progression of DN [11], involving a complex network of molecules and biological processes [12, 13]. For example, IL-8 is increased in the serum of type 2 diabetic patients and causes damage to podocytes via the IL-8-CXCR1/2 axis, further exacerbating renal damage in the presence of other toxic and proinflammatory factors [14, 15]. CXCR1/2 can act as a receptor for several CCL family chemokines and promote the accumulation of multiple immune cells (e.g., macrophages and neutrophils) to sites of inflammation [16]. In contrast, blocking the IL-8-CXCR1/2 axis improves renal function and reduces thylakoid expansion in diabetic mice [17]. In addition, some chemokines and chemokine receptors (CCRs) were also found to show an increase in glomeruli and proximal tubules in DN animal models [18, 19]. Among them, CCL2 is one of the key CCRs involved in regulating the recruitment of monocytes, macrophages, T cells, and dendritic cells to sites of inflammation [10]. In animal models of diabetes-induced kidney injury, the expression of CCL2 is significantly increased [20]. By using CCL2 inhibitors, the urinary albumin-creatinine ratio can be reduced [21]. And inhibition of CCL2 expression also reduces the migration of activated macrophages, which in turn reduces renal fibrosis [22].

Currently, no study has reported the interaction between CCRs and immune cells during the progression of DN to ESRD. Therefore, this study analyzed differentially expressed genes (DEGs) in DN and ESRD patients by bioinformatics. The relevant biological pathways were identified by enrichment analysis. A protein-protein interaction (PPI) network was established using DEGs, identifying hub genes, intersecting them with CCRs, and discovering hub CCRs. Significantly altered immune cells were identified using immune infiltration analysis, and correlations between immune cells and hub CCRs were analyzed. The aim of this study was to explore the hub CCRs that play a key role in the progression of DN to ESRD and their impact on the immune microenvironment, providing new targets for the prevention and treatment of ESRD.

## 2. Methods

### 2.1. Microarray Data Sources

The datasets were downloaded from the Gene Expression Omnibus database. Both DN and ESRD gene expression data were obtained from GSE142153 [23]. The data included 23 DN samples and 7 ESRD samples. The peripheral blood of the samples was analyzed with the platform number GPL6480. This gene set data has been normalized.

### 2.2. Identifying DEGs

After normalization and preprocessing of the data, the probes were annotated. This is done through the “GEOquery” package of R software. If multiple probes correspond to the same gene, only the probe with the highest average expression is retained. After completion of probe annotation, the “limma” package was used to screen for differentially expressed genes (DEGs). Genes with *P* < 0.05 and |log fold change (FC)| > 1 were DEGs for DN and ESRD. The screening results are shown by volcano plot.

### 2.3. Enrichment Analysis

Enrichment analysis was performed using DEGs, including KEGG pathway enrichment analysis and GO enrichment analysis (biological process (BP), cell composition (CC), and molecular function (MF)). All were performed through the Metascape database (https://metascape.org/). Limiting species were Homo sapiens, min overlap ≥ 3, min enrichment ≥ 1.5, and *P* < 0.01 as threshold.

### 2.4. Construction of PPI Network and Identification of Hub Genes

Import the DEGs into the STRING database to construct the PPI network. Import the results of the analysis into Cytoscape software for visualization. The core targets of the PPI network were analyzed using CytoHubba plug-in. The top 10 targets with MNC, MCC, degree, and closeness scores were calculated separately [24]. The intersection of the four scores was hub genes. The results were presented using the Venn diagram.

### 2.5. Obtaining Hub CCRs

CCRs were derived from studies by others [25]. The Venn diagrams were used to obtain intersectional targets of hub genes with CCRs that are hub CCRs. The expression of hub CCRs in different subgroups was demonstrated using box line plots. The diagnostic value of hub CCRs was analyzed using ROC curves. Genes with AUC > 0.7 were considered to have a high diagnostic value.

### 2.6. ssGSEA of Immune Infiltration

The degree of infiltration of 28 immune cell species in all samples was analyzed using the ssGSEA algorithm. This method allowed to obtain the relative expression abundance of immune cells. The differences of each immune cell in different groups were analyzed according to the grouping. In addition, correlations between immune cells were calculated using the Spearman correlation. In combination with hub CCRs, the Spearman correlations between hub CCRs and different immune cells were calculated. The above results were plotted using the “ggplot2” program package.

### 2.7. Statistical Analysis

Comparisons between groups were analyzed for differences in nonnormally distributed data using the Wilcoxon rank sum test. Correlation scores were calculated using Spearman's method. *P* < 0.05 was considered statistically different.

## 3. Results

### 3.1. Screening DEGs and Building PPI Networks

**Figure 1:**
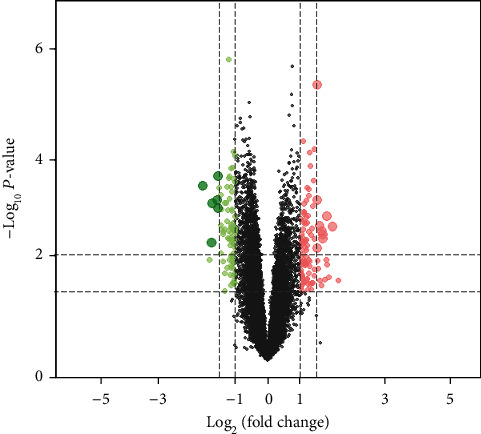
Volcano plot of differentially expressed genes in DN patients and ESRD patients. The dashed line on the *x*-axis corresponds to |FC| = 1 or 1.5. The dashed line on the *y*-axis corresponds to *P* = 0.05 and 0.01. Green indicates downregulated DEGs. Red indicates upregulated DEGs. DEGs: differentially expressed genes; DN: diabetic nephropathy; ESRD: end-stage renal disease; FC: fold change.

According to the set screening criteria, *P* < 0.05 and |log FC| > 1, a total of 181 DEGs were obtained after screening. Among them, there were 99 upregulated DEGs and 82 downregulated DEGs. The volcano plot of DEG distribution is shown in Figure 1.

### 3.2. Biological Pathway Enrichment Analysis

**Figure 2:**
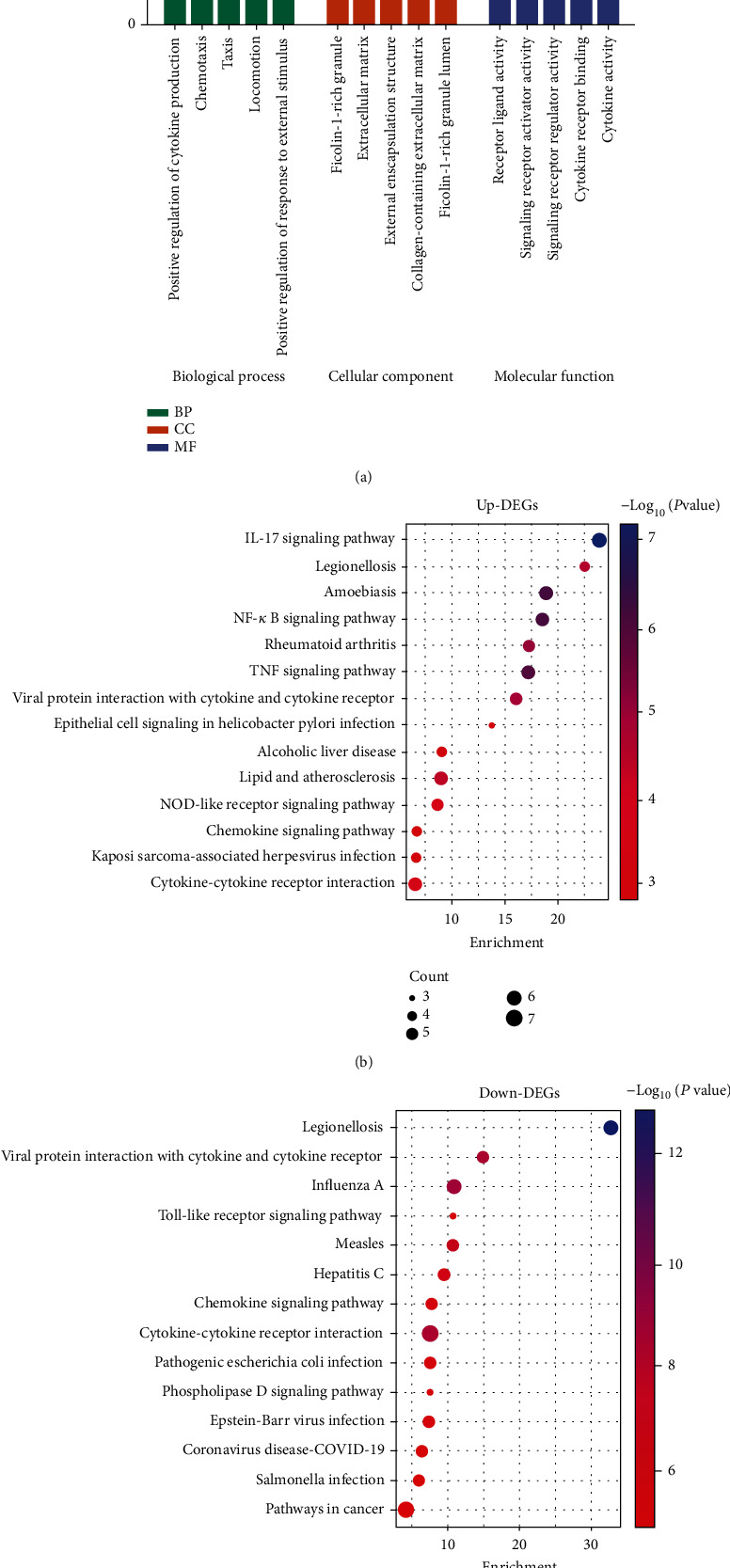
Enrichment analysis results. (a) GO enrichment analysis. Different colors indicate different classifications. The *x*-axis is the term name, and the *y*-axis is the enrichment fraction. (b, c) KEGG enrichment analysis of upregulated/downregulated DEGs. The *x*-axis is the enrichment analysis. The *y*-axis is the pathway name. The size of the bubbles indicates the number of genes annotated to the pathway. The color of the bubbles indicates the *P* value.

The results of the GO enrichment analysis are shown in Figure 2(a). The top 5 terms in each section are shown. The results show that biological processes such as cytokines, signaling receptors, and extracellular matrix are involved in the disease process from DN to ESRD. KEGG pathway enrichment analysis was performed using upregulated/downregulated DEGs separately, showing the top 14 pathways in terms of *P* value, respectively (Figures 2(b) and 2(c)). Among the enrichment results of upregulated DEGs, IL-17 signaling pathway, NF-kappa B signaling pathway, TNF signaling pathway, NOD-like receptor signaling pathway, cytokine-cytokine receptor interaction, and chemokine signaling pathway may be activated during disease progression. In the enrichment results of downregulated DEGs, cytokine-cytokine receptor interaction, chemokine signaling pathway, Toll-like receptor signaling pathway, and other pathways may be activated. The above results suggest that CCRs may be one of the key factors in the progression of DN to ESRD.

### 3.3. Construction of PPI Network and Identification of Hub Genes

**Figure 3:**
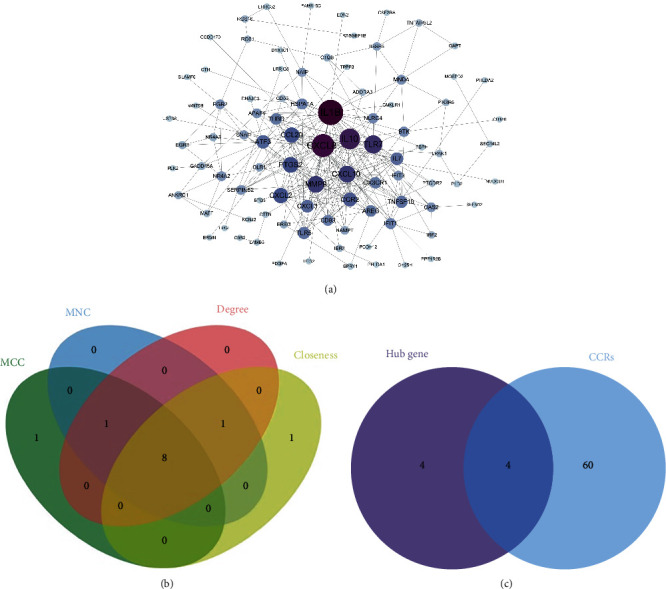
Construction of PPI networks and identification of hub genes and hub CCRs. (a) PPI network constructed using DEGs. Each point represents a DEG. The size of the point and the shade of the color correspond to the size of the degree value. (b) The hub genes were screened by four algorithms (MCC, MNC, degree, and closeness). (c) Intersecting hub genes with CCRs to identify hub CCRs. CCRs: chemokines and chemokine receptors; PPI: protein-protein interaction.

**Figure 4:**
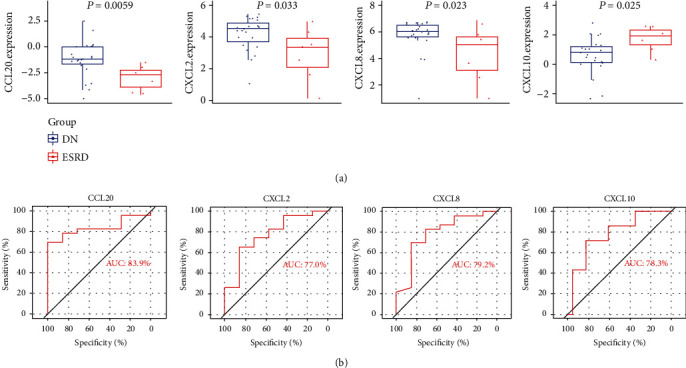
The expression profile and diagnostic value of hub CCRs. (a) The expression of hub CCRs is shown using box line plots. Blue indicates DN patients, and red indicates ESRD patients. (b) ROC analysis of hub CCRs. Larger AUC indicates higher diagnostic value. AUC: area under the curve; ROC: receiver operating characteristic curve.

PPI networks are created using the STRING database. The generated network has 161 nodes, 261 edges, an average degree of 3.24 per node, and PPI enrichment *P* value < 1.0*e*-16. The results were imported into Cytoscape software for visualization (Figure 3(a)). Using CytoHubba plug-in to calculate MNC, MCC, degree, and closeness scores and taking their intersection, a total of 8 hub genes were found: CXCL8, MMP9, IL1B, TLR7, CXCL10, IL10, CXCL2, and CCL20 (Figure 3(b)). A total of 64 CCRs were obtained from previous studies (Table 1), and a total of 4 hub CCRs were obtained by intersecting CCRs with hub genes: CXCL2, CXCL8, CXCL10, and CCL20. These hub CCRs may act as core regulatory targets in disease progression from DN to ESRD and have an impact on the disease. Box plots of the expression of hub CCRs in different subgroups are shown in Figure 4(a). ROC analysis of hub CCRs showed that four hub CCRs had AUC > 0.7, indicating that they all have high diagnostic value and can be used to differentiate DN from ESRD (Figure 4(b)).

### 3.4. Immunoinfiltration Analysis

**Figure 5:**
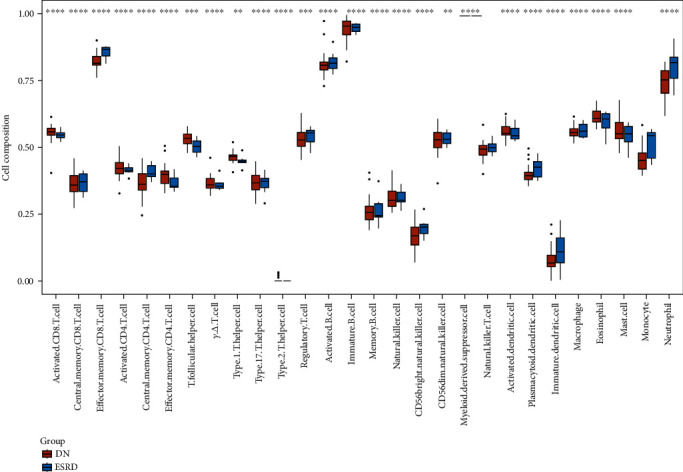
Immune cell expression box plot. Different colors indicate different groups. ^∗∗^*P* < 0.01, ^∗∗∗^*P* < 0.001, and ^∗∗∗∗^*P* < 0.0001.

**Figure 6:**
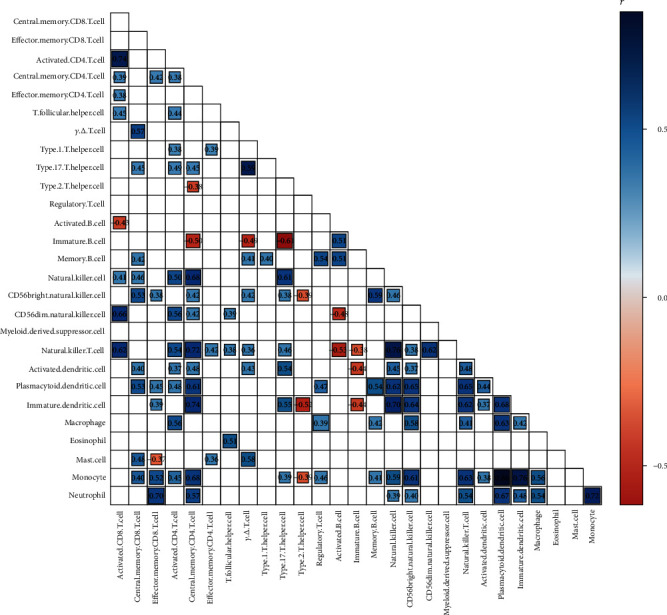
Immune cell correlation graph. Each square indicates whether there is a correlation between two immune cells. The number in the box indicates the correlation value. The color of the box corresponds to the correlation value.

Using the ssGSEA method, the degree of immune cell infiltration was calculated in all samples. When using the ssGSEA method, the expression abundance of different immune cells was mapped to 0-1 depending on their expression. The relative expression abundance of myeloid-derived suppressor cells was highest in each sample. Therefore, the relative expression abundance of this cell was 1 in each sample, and in the between-group analysis, the variance was 0. In Figure 5, the relative expression of various immune cells in different groups is shown. In DN versus ESRD, most of the immune cells were significantly changed. Immune cells with significantly higher infiltration in ESRD samples were effector memory CD8 T cells, central memory CD4 T cells, regulatory T cells, activated B cells, CD56bright natural killer cells, natural killer T cell, plasmacytoid dendritic cell, immature dendritic cell, macrophage, monocyte, and neutrophil. In Figure 6, the correlations between different immune cells are shown. Among them, the |correlation coefficients| ≥ 0.7 are 0.74 (activated CD8 T cell with activated CD4 T cell), 0.70 (effector memory CD8 T cell with neutrophil), 0.72 (central memory CD4 T cell with natural killer cell), 0.74 (central memory CD4 T cell with immature dendritic cell), 0.76 (natural killer T cell with natural killer cell), 0.70 (natural killer cell with immature dendritic cell), 0.86 (plasmacytoid dendritic cell with monocyte), 0.76 (immature dendritic cell with monocyte), and 0.72 (monocyte with neutrophil).

### 3.5. Correlation Analysis of Immune Cells and Hub CCRs

**Figure 7:**
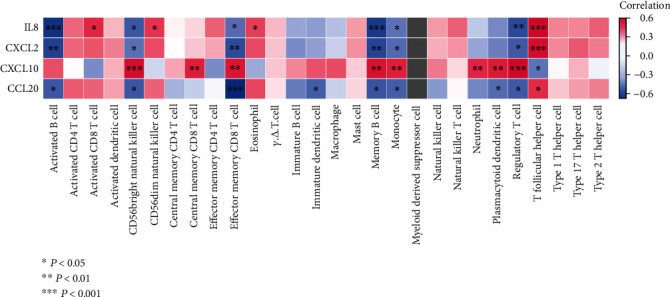
Correlation of immune cells with hub CCRs. Each square indicates the correlation of hub CCRs with immune cells. Different colors correspond to different correlation values. ^∗^*P* < 0.05, ^∗∗^*P* < 0.01, and ^∗∗∗^*P* < 0.001.

To further elucidate the correlation between hub CCRs and immune cells, the Spearman correlation coefficient between hub CCRs and immune cells was calculated (Figure 7). The results showed that the immune cells that were significantly correlated with four hub CCRs were CD56bright natural killer cell, effector memory CD8 T cell, memory B cell, monocyte, regulatory T cells (Tregs), and T follicular helper cell. It indicates a higher degree of influence on these immune cells during the regulation of disease by hub CCRs. They may play a regulatory role in the process of DN to ESRD.

## 4. Discussion

In the present study, DEGs were identified in the progression of DN to ESRD. Through enrichment analysis, chemokine signaling pathways, cytokine-cell receptor interaction pathways, and inflammation-related pathways were found to play important roles in the development of DN patients. We then established a PPI network to obtain hub genes, crossed them with CCRs, and identified four hub CCRs. The results of immune infiltration showed a correlation between hub CCRs and various immune cells. Most importantly, CCRs may be involved in the process from DN to ESRD by regulating the immune environment.

KEGG enrichment analysis was divided into upregulated DEGs and downregulated DEGs. The enrichment results of upregulated DEGs include chemokines, cytokines, and inflammation-related pathways. NF-*κ*B is a key intracellular molecule for monitoring inflammatory response signals, and when NF-*κ*B is activated, it promotes transcription of inflammatory cytokines (e.g., TNF-*α*, IL-1*β*, and IL-6), which is highly correlated with DN progression [26, 27]. By inhibiting NF-*κ*B signaling, macrophage infiltration into the kidney can be suppressed, and the expression of TNF-*α*, IL-1*β*, and MCP-1 can be reduced [26]. IL-17A is a cytokine that is produced by several types of cells, such as T cells, natural killer cells, neutrophils, macrophages, and dendritic cells [28]. IL-17A is involved in tissue inflammation by inducing the expression of CCRs, inflammatory cytokines, and matrix metalloproteinases (MMPs) [29]. Inhibition of IL-17A may be a key factor in the prevention of ESRD [30]. The relevance of CCRs to DN has been demonstrated. Patients with DN have enhanced expression of CXCL9 in serum and urine compared to healthy populations [31]. CXCL7 from platelet particles causes glomerular endothelial injury, which is significantly attenuated when CXCL7 receptors are blocked with competitive CXCR1/2 inhibitors [32]. CCL2 can attract or enhance the expression of inflammatory cytokines, reflecting tubular injury and renal inflammation in DN [33–35]. In addition, CCR2 has been shown to cause podocyte loss and apoptosis in DN [36]. Meanwhile, clinical trials have found that CCL2 levels in urine are a key biomarker for predicting rapid decline in renal function in DN patients [37]. The enrichment of downregulated DEGs resulted in enrichment of pathways such as chemokines, cytokines, and Toll-like receptor (TLR) signaling pathway. TLRs are a superfamily of innate immune system receptors that monitor and recognize exogenous invading pathogens to accelerate the immune response [38]. TLRs are expressed on a variety of cells, including antigen-presenting cells and renal lamina propria, and when TLRs are activated, they exacerbate the inflammatory response and lead to the progression of DN [39]. Feng et al. showed that the expression levels of TLRs were significantly upregulated in a porcine model of DN, which subsequently activated the downstream NF-*κ*B and IRF-3 signaling pathways, ultimately leading to increased mRNA expression of IL-6, CXCL2, and CCL5 [40]. In addition, TLR4/NF-*κ*B signaling pathway was activated in DN mice, resulting in increased expression of CCL2, CCL20, CXCL5, and CXCL7 [18]. GO enrichment analysis revealed that biological processes such as cytokines, signaling receptors, and the extracellular matrix (ECM) may be involved in the progression of DN to ESRD. MMPs, especially MMP-2 and MMP-9, affect ECM catabolism and turnover [41]. It has been found that MMP-2 and MMP-9 expressions are dysregulated in both acute and chronic kidney diseases [42]. Interestingly, the CXCL8 antagonist G31P has been shown to improve renal fibrosis by reducing ECM, which may be associated with improved expression of MMP-2 and MMP-9 [43]. In the results of GO and KEGG enrichment analyses, CCRs were found to possibly play a key role in the progression of DN to ESRD.

To further elucidate the role of CCRs in the progression of DN to ESRD, a PPI network was constructed using DEGs and 8 hub genes were identified. In combination with CCRs, a total of 4 hub CCRs were identified (CXCL2, CXCL8, CXCL10, and CCL20). These hub CCRs are members of the CXC and CC major subfamilies and can function as signaling molecules that bind to chemokine receptors on the cell surface to produce immunosurveillance. CXCL2 (MIP-2), a member of the mouse CXC family, is a functional analogue of human CXCL8 [44]. CXCL2 recruits neutrophils and activates neutrophils during inflammation [45]. The expression levels of this chemokine were not consistent in diabetes-related studies [46, 47], and the reasons for the discrepancy may be related to the spectrum of target cells and stimulation. In their study, Marisa et al. found that hypoxia, associated with reduced MIP-2 and increased MIP-1, plays a key role in the development of diabetic complications. Reduced MIP-2 may lead to a shortened functional life span of neutrophils with reduced infiltration, disturbed cytokine production, and increased susceptibility to infection [48]. Then, there is a trend towards increased expression of MIP-2 in aging diabetic-tethered cells [49]. Meanwhile, in the pathological state of kidney damage, there are proinflammatory tubular cells that are characterized by increased expression of CXCL2, which may be closely related to ESRD [50]. CXCL8 (IL-8) was originally discovered and purified as a chemokine for neutrophils [51]. Nowak et al. found that urinary IL-8 levels were higher in patients with DN and, moreover, the highest levels were found in patients with the lowest glomerular filtration rate and the poorest renal function [37]. In addition, excessive activation of the IL-8-CXCR1/2 axis is responsible for podocyte damage or loss in DN [16]. The same results were confirmed in vivo, where blockade of the IL-8-CXCR1/2 axis by CXCL8 antagonists produced beneficial effects on renal function [17, 43]. In ESRD patients on dialysis, if CXCL8 exhibits significant retention with CCL2, it can lead to poor clinical outcomes [52, 53]. CXCL10 exerts its biological functions mainly through binding to its specific receptor CXCR3. Elevated levels of CXCL10 in serum have been reported in diabetic patients [54–56]. CXCL10 is a chemoattractant for leukocytes and also has a vasopressor effect that leads to disease progression and worsening prognosis in CKD patients [57]. Schulthess et al. found that CXCL10 can act as a binding partner for TLR4 and activate the Akt-JNK-PAK2 pathway to promote the signaling switch from proliferation to apoptosis in *β* cells of diabetic patients [56]. Furthermore, damaged tubules will express high levels of CXCL10 when mice have more severe inflammation, which further increases the risk of CKD [58]. CCL20 is the only chemokine known to bind to CCR6 and drive the migration of CCR6 cells in tissues [59]. CCR6 is expressed in a variety of immune cells, including B cells, immature dendritic cells (DCs), regulatory CD4 T cells, and T helper cells 17 (Th17 cells), and is involved in cell migration under physiological and inflammatory conditions [60–62]. It has been shown that blocking the binding of CCR6 to CCL20 and inhibiting the expression of CCR6 on immune cells reduce islet infiltration and inhibit the development of diabetic mice [63]. Marisa et al. found that CXCL2 was downregulated in diabetic rats under hypoxic conditions [48]. In addition, targeting CCL20 could alleviate renal fibrosis through regulating fibroblast proliferation and suppressing collagen I expression [64].

By immune cell infiltration analysis, we found that a variety of immune cells changed significantly during the course of disease changes and that there was a significant correlation between some immune cells and hub CCRs. Chronic inflammation is a major factor in the progression of DN [65], and the degree of immune cell infiltration, especially macrophages, is directly related to the severity of DN [20, 66]. Macrophages are the most prevalent infiltrating leukocytes in DN and are involved in the decline of renal function in DN [67]. It has been found that mice lacking the CCL2 gene do not develop ESRD because of their reduced ability to recruit and activate macrophages [20, 68]. DCs are a type of antigen-presenting cells that are essential for the initiation and regulation of the immune response [69]. It has been reported that circulating DCs are significantly reduced in ESRD patients, especially plasmacytoid DCs [70]. In addition, macrophages and DCs stimulated by high glucose levels prompt the differentiation of naive T cells into Th17 cells that can produce IL-17A. Th17 cells recruit and activate innate immune cells, while stimulating proximal tubular HK-2 cells to produce CCL20. CCL20 leads to migration of macrophages and lymphocytes to surrounding tissues, further aggravating renal injury [71]. Elevated T cells in the kidney are also associated with the progression of DN [12]. CD4+ T cells and CD8+ T cells infiltrate significantly in the renal interstitium of STZ-induced diabetic mice and can release IFN-*γ* and TNF-*α*, activate macrophages and endothelial cells, and promote inflammation [72]. IL-17A expressed by CD4 T cells is associated with an increase in inflammatory cytokines, macrophage infiltration, and increased renal damage. *In vitro*, inhibition of IL-17A protects podocytes and inhibits NF-*κ*B activation, thereby ameliorating DN progression [73]. Tregs are key cells that regulate inflammation and maintain immune self-tolerance and homeostasis [74]. ESRD patients are characterized by an overactivated but impaired immune system, which may be caused by Treg dysfunction and reduced numbers [75]. In diabetic mice, Treg depletion leads to severe DN and exhibits increased albuminuria [76]. This is similar to what is seen in ESRD patients in the clinic [77]. In addition, an increased ratio of Th17/Tregs was positively correlated with the severity of CKD [78]. The above results suggest that CCRs are capable of influencing immune cells during the progression of DN to ESRD, which may contribute to the disease progression.

## 5. Conclusion

In brief, this study shows the key mechanisms of DN progression to ESRD. Enrichment analysis revealed that chemokine-related pathways are involved in disease progression. By constructing a PPI network, four hub CCRs were identified that play a key role in the progression of DN to ESRD. In addition, multiple immune cells underwent significant changes and were associated with hub CCRs. The above results suggest that the immune environment influenced by chemokines may contribute to the progression of DN to ESRD.

## Figures and Tables

**Table 1:** All CCRs. CCRs: chemokines and chemokine receptors.

CCRs			
CCL1	CCL19	CXCL6	CCR3
CCL2	CCL20	CXCL7	CCR4
CCL3	CCL21	CXCL8	CCR5
CCL3L1	CCL22	CXCL9	CCR6
CCL4	CCL23	CXCL10	CCR7
CCL4L1	CCL24	CXCL11	CCR8
CCL5	CCL25	CXCL12	CCR9
CCL7	CCL26	CXCL13	CCR10
CCL8	CCL27	CXCL14	CXCR1
CCL11	CCL28	CXCL16	CXCR2
CCL13	CXCL1	CXCL17	CXCR3
CCL14	CXCL2	XCL1	CXCR4
CCL15	CXCL3	XCL2	CXCR5
CCL16	CXCL4	CX3CL1	CXCR6
CCL17	CXCL4L1	CCR1	XCR1
CCL18	CXCL5	CCR2	CX3CR1

## Data Availability

In this study, publicly available datasets were analyzed, which can be found at https://www.ncbi.nlm.nih.gov/geo/.
